# The impact of an integrated nurturing care intervention to improve early childhood development outcomes in Nampula Province, Mozambique

**DOI:** 10.1017/S1368980025100554

**Published:** 2025-06-13

**Authors:** Filipa de Castro, Katija Momade, Jennifer Yourkavitch, Charles D. Arnold, Alberto Manhiça, Filipe Zano, Higino André, Edmilson Ismail, Kristen Cashin, Catherine M. Kirk

**Affiliations:** 1 Save the Children US, Fairfield, CT, USA; 2 USAID Advancing Nutrition Mozambique, Nampula, Mozambique; 3 Save the Children Mozambique, Nampula, Mozambique; 4 USAID Advancing Nutrition, Arlington, VA, USA; 5 Results for Development, Washington, DC, USA; 6 University of California Davis, Davis, CA, USA; 7 Ajuda de Desenvolvimento de Povo para Povo (ADPP), Nampula, Mozambique; 8 Transform Nutrition, Nampula, Mozambique; 9 ZemiTek LLC, USAID’s Global Solution Ventures, Washington, DC, USA

**Keywords:** Nutrition and caregiving interventions, Early childhood development, Nurturing care, Early learning, Early stimulation

## Abstract

**Objective:**

To estimate the effect of integrating responsive care, early learning and development monitoring into a community-based package of activities on nutrition, sanitation and hygiene, on improvements in early childhood development (ECD) outcomes.

**Design:**

This was a quasi-experimental study with nonequivalent comparison groups. The study’s primary outcome, ECD, was measured using the Ages and Stages Questionnaire (ASQ-3) and the Global Scales for Early Development (GSED). We also collected data on the early learning home environment, nutritional practices and caregiver depressive symptoms as secondary outcomes.

**Setting:**

This study was conducted across twelve districts in Nampula Province, Mozambique. Half of the districts received holistic nurturing care with responsive care, early learning, nutrition, sanitation and hygiene packages (intervention) and the other half received only nutrition, sanitation and hygiene packages (comparison).

**Participants:**

We recruited an age-stratified random sample of 961 caregivers and their children, aged 0–23 months.

**Results:**

We found a significantly higher mean caregiver engagement total score (mean difference: 0·36; *P* ≤ 0·001) and a higher number of activities to support learning (mean difference: 0·30, *P* = 0·004) in the intervention group than in the control. There were no measurable impacts on the remaining early stimulation activities or the primary outcomes of the ASQ and GSED developmental scores.

**Conclusions:**

We discuss the challenges in the integration of nurturing care interventions into existing programmes in high-vulnerability contexts, highlighting the aspects needed to achieve effective caregiver behavioural changes that can translate into improved ECD outcomes.

Nurturing family environments that promote health, nutrition and early learning opportunities, nested in emotionally supportive and safe relationships are fundamental to provide children with an optimal start in life^(1)^. When established during early childhood, these opportunities can mitigate the risk of poor outcomes arising from inequalities, poverty and other contextual and environmental threats^(2,3)^. Opportunities for early childhood development (ECD) are influenced by the child’s immediate surroundings (e.g. parents, siblings and caregivers), immediate settings (e.g. home and community environments) and external systems (e.g. early childhood services and policies)^(4,5)^.

There is a growing body of evidence supporting the implementation of holistic ECD interventions integrated into and delivered through existing, cross-sectoral platforms^(6,7)^. Evidence from low- and middle-income countries indicates that services combining caregiving and nutrition interventions provide greater benefits for improving children’s cognitive, language and motor development, compared with single-component interventions^(8)^. Further, multi-sectoral interventions are well understood as essential for making progress to reduce undernutrition^(9)^. In this context, the Nurturing Care (NC) framework, globally promoted by the WHO, United Nations Children’s Fund, World Bank Group and partners^(10)^, recommends the deployment of integrated interventions that target systems, communities, caregivers and children to promote children’s health, growth and development during infancy and early childhood. Multi-sectoral interventions delivering multiple inputs have been recognised to improve child growth and development in low- and middle-income countries (LMIC), being preferred to interventions providing only individual inputs^(11)^. Evidence suggests that interventions combining responsive care and enhanced nutrition can leverage mechanisms from both nutrition and mother–child interactions and are likely to be more effective at improving child outcomes than interventions promoting only one of the mechanisms^(12,13)^. However, findings around improved growth outcomes are mixed in both integrated programmes, as well as single-component nutrition programmes for young children that focus only on the promotion of infant and young child feeding practices^(8,14)^. In other words, mother–child interactions are an important mediator of the effect of responsive stimulation and nutrition interventions. Furthermore, more attention needs to be paid to the resources caregivers require – including their health and well-being – to provide nurturing care that supports optimal growth and development ^(15)^.

While there is strong evidence based on integrated approaches, research has shown mixed results in LMIC often due to implementation challenges^(16,17)^. Implementing evidence-based ECD programmes is complex and involves substantive adaptations to local contexts^(18)^ with varying effects on child development outcomes, even when the same curriculum is followed^(6)^. With the growing endorsement and demand for integrated programmes to improve ECD, evidence is needed on how to deliver them effectively through existing platforms and systems. More understanding is needed on the benefits and feasibility of leveraging the touchpoints of existing services, including maternal and child health and nutrition services, with caregivers and children during the first 1000 d.

In this study, we report whether and how the integration of additional NC activities into an existing community-based nutrition platform in Mozambique has changed caregiver practices and ECD outcomes. There is a strong rationale to promote NC interventions in the country, where 72 % of young children are at risk of poor development^(19)^, with a large proportion experiencing chronic undernutrition, poor early nutritional practices and lack of stimulating learning environments^(20)^. Mozambique’s fragile health and nutritional situation has been aggravated by recent events, including conflicts in the north of the country, climate shocks (cyclones and tropical storms in the centre and north and drought in the south), residual impacts of the COVID-19 pandemic and the impact of global prices of food and essential nutritional products due in part to the war in Ukraine^(21)^.

Building on the government’s momentum in promoting NC to improve ECD through health and nutrition services, we implemented a structured caregiving intervention in Nampula Province in northern Mozambique. Nampula is the most populated province in the country, with close to 6·5 million inhabitants, two-thirds of whom live in rural areas. There are close to one million children under 5 years of age in Nampula, comprising approximately 15 % of the province’s population^(22)^. Children in Nampula experience some of the worst living conditions in the country. More than half of the households do not have access to a safe water source; the prevalence of stunting among children under the age of five is 46·7 %, and seven out of every 100 children born alive die before reaching the age of five^(21)^. Our intervention complemented the existing community-based nutrition programme, which was delivered through the USAID-funded Transform Nutrition Activity to support the Government of Mozambique’s nutrition intervention package (Pacote de Intervenções de Nutrição) and Multi-sectoral Plan of Action for the Reduction of Stunting (Plano de Acção Multissectorial para a Redução da Desnutrição Crónica em Moçambique), with a package to promote holistic NC. The objective of this study was to examine the impact of the holistic NC package delivered through frontline community-based workers and volunteers on children’s developmental status, as the study’s primary outcome. The study also examined the quality of early learning at home, infant and young child feeding practices and caregiver psychosocial well-being, as secondary outcomes.

## Methods

### Population and study settings

We conducted a quasi-experimental study with a non-equivalent comparison group across twelve districts in Nampula.

**Figure 1.**
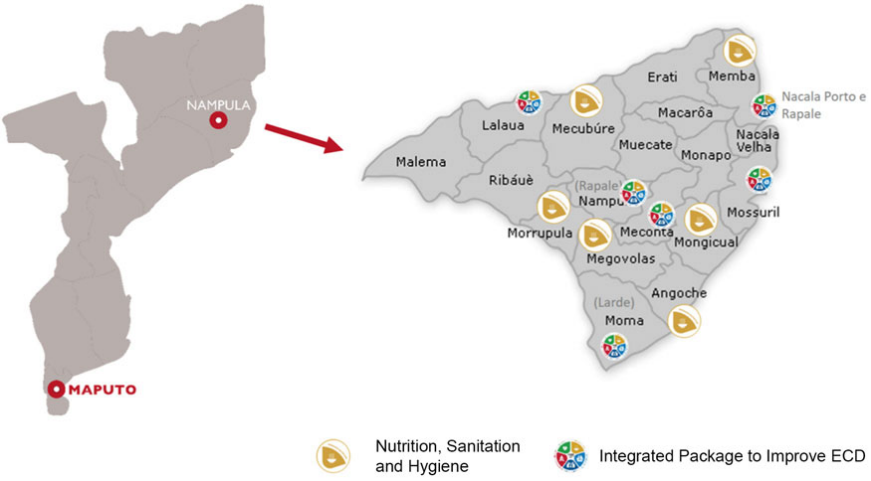
Intervention and comparison districts.

We assigned districts to the intervention or comparison group following a feasibility approach, with smaller districts allocated to the intervention by the local implementing partner for ease of programmatic logistics alongside other implementation research activities. Six comparison districts (Mecubure, Memba, Murrupula, Mongicual, Angoche and Mogovolas) received a community-based package of nutrition, sanitation and hygiene (SH) interventions. Six intervention districts (Nacala-Porto, Larde, Lalaua, Rapale, Mossuril and Meconta) received the same nutrition and SH intervention complemented by responsive care, early learning and monitoring children’s development to improve ECD outcomes, referred to as the NC package. (Figure 1)

### Intervention description

We integrated the holistic NC package into an existing programme platform for nutrition and SH activities delivered through the USAID-funded activity Transform Nutrition^(23)^. In the nutrition and SH intervention, frontline community-based staff, including community health workers, health promoters and supported volunteer nutrition group leaders, delivered nutrition services and promoted social and behavioural change interventions organised around nutrition groups. Nutrition group leaders who facilitated the groups and conducted home visits were volunteers who were recruited from the same community and were expected to have basic literacy and numeracy skills. Using a Portuguese language manual appropriate for caregiver group interventions, nutrition groups learn and exchange knowledge and skills while following a structured programme that emphasised interactive activities, direct practice, feedback and reflection. The groups were facilitated by nutrition group leaders on a weekly basis (1·5–2 h each) over the course of about 7–8 months for groups of 10 caregivers with their children ages 0–2 years, primarily mothers, with about five community influencers including male caregivers, local leaders and others. The nutrition group leaders also conducted monthly home visits for follow-up with caregivers who participated in the community group, focusing on those who needed additional support. Community radio and video messaging, in the local language of Makhuwa, are also used to broadcast additional content at this scale.

We embedded our NC activities into each of the nutrition and SH streams of activities described above and included promoting responsive care, early learning and development monitoring through community nutrition groups, home visits and radio/video spots, as detailed in Table 1. The additional components were adapted from previous studies on promoting ECD through primary healthcare in Mozambique^(24)^. We used a cascade training approach, guided by principles of adult learning, with follow-up supervision to strengthen the capacity of community-based workers to deliver the expanded NC content and support the workers to be able to successfully deliver the integrated approach. The initial training of trainers was conducted in Portuguese for district-level staff and area leaders who oversaw a cluster of community health workers and their associated nutrition group leaders. Trainees were evaluated using pre–post knowledge assessments and supervision checklists to assess the application of core skills during group sessions. Data from these tools were published elsewhere^(25)^. The local implementing partner’s mentors supervised the nutrition group leaders and supplemented them with routine visits from the USAID Advancing Nutrition Team.

**Table 1.** Description of intervention and comparison groups and of the intervention contents and activities

Implementation setting	Comparison	Intervention
Number of districts	6	6
Number of target communities	991	539
Target population	78 780	39 135
Intervention platform	Ongoing multi-programme nutrition platform	
Service providers	Community nutrition group leaders are supported by community health workers and health promoters	
Number of service providers	529	296
Number of caregivers at baseline	485	476
Intervention content and activities	Comparison	Intervention
Community group sessions	Twenty-six community group sessions on nutrition, sanitation and hygiene delivered weekly	
		Opening play-based activity (song or game) for all sessions
		Four additional sessions with play-based activities linked to the session’s main theme
Content of group sessions	Maternal health and nutrition in pregnancy, exclusive and continued breastfeeding, complementary feeding, family vegetable garden and small animal husbandry	
	Nutrition in special situations	
	Sanitation and hygiene	
		Responsive care (e.g. recognising and responding to a child’s cues) and early learning (e.g. engaging in play with children including during daily routines and communicating with children starting before birth)
		Supporting and monitoring child development using milestones on the child health card
Health and growth monitoring and promotion	Health promotion, growth monitoring, micronutrient supplementation	
		Developmental screening and referral
Home visits	Monthly home visits focused on challenges and problem-solving around nutrition practices and encouraging the production of homemade toys and play with household objects	
Mass media through radio and community video groups	Community radio services in Makhuwa language	
	Community video groups delivered through video trucks, involving the presentation and moderated discussions of locally produced videos in Makhuwa language, addressing best practices around nutrition topics	
		Additional video content focusing on responsive care and early learning behaviours included
		Broadcasting on ECD topics through community radio services

The materials for nutrition group volunteers and community health workers were provided in Portuguese, which is consistent with the approach of the Ministry of Health. Mass media radio and video spots were developed in the local language, most predominantly spoken in Nampula Province, Makhuwa.

ECD, early childhood development.

### Sample design and recruitment

We identified participants in the twelve districts through Transform Nutrition programme documents on nutrition and SH interventions. Our sampling frame comprised all caregivers and their children under 24 months of age who had been identified for community-based activities from among all communities in each district. We considered primary caregivers, defined as the person providing daily care for the child, for inclusion if they were enrolled in the community groups of the nutrition and water, sanitation and hygiene intervention platform and were aged 18 years or older.

Following a cluster sample two-stage design, we first selected a sample of forty-seven communities in each district using a probability proportional to size. In the second stage, we randomly selected ten caregiver–child dyads from a list for each selected community stratified by child’s age at baseline. We aimed to obtain an equal distribution of children across six age brackets at baseline (0–2, 3–5, 6–8, 9–11, 12–17 and 18–23 months).

For each community, we followed a consistent process for informing the intervention community health workers about the selected households and seeking consent from the household prior to the completion of the interviews and assessments.

#### Sample size, power and detectable difference

Our sample size targets the detection of a change of 0·3 sd in overall child development, in line with a medium effect size from several systematic reviews of parenting interventions^(7,26)^ and assumes a design effect of 2 to adjust for clustering of caregivers in the nutrition groups (i.e. some of the caregiver–child dyads will come from the same nutrition groups), alpha of 0·05, power of 80 % and a 25 % loss to follow-up between the baseline and endline assessments.

Thus defined, we calculated that a sample of 930 caregiver–child dyads, 465 in each of the intervention and comparison groups, was required to detect differences in child development outcomes between the two groups.

#### Baseline and endline data collection

We collected baseline data from February to March 2022, and endline data collection took place 1 year later, from February to March 2023. Data were collected across one or two days per participating household using tablet-based data collection software. All supervisors and enumerators received standardised training on the content and intent of the survey questions, data collection software, approach to ensure ethical, secure and private data collection, as well as the process and requirements of informed consent. A validation process was completed for administration of the direct observation Global Scales for Early Development (GSED) tool. Additional details on enumerator training and evaluation are published elsewhere^(27)^. We monitored the fieldwork progress and data quality indicators throughout the duration of data collection. Neither the data collectors nor the supervisors were blinded to the intervention assignments

#### Study outcomes and data collection tools

We administered eight tools to primary caregivers to collect data on the socio-demographic characteristics of the caregiver and the child, household assets and wealth, overall child development, early learning environment at home, infant and young child feeding practices and caregiver’s mental health.

### Developmental status

Our primary outcome of interest was measured by the caregiver reported on the Ages and Stages Questionnaire^(28)^ (ASQ-3), previously used in Mozambique^(29)^. ASQ-3 consists of age-specific forms, each with thirty questions that capture development across five domains: fine motor, gross motor, communication, problem-solving and personal–social skills. In this study, we used 12 different ASQ-3 forms at baseline (the 2-month, 4-month, 6-month, 8-month, 10-month, 12-month, 14-month, 16-month, 18-month, 20-month, 22-month and 24-month forms) and four additional ASQ-3 forms at the end line (the 27-month, 30-month, 33-month and 36-month forms). We analysed ASQ-3 as a continuous score, standardised to in-sample sd units, using the standardised residuals from a linear regression of ASQ-3 on age, sex and the data collector. This approach is analogous to the length-for-age z-score, in that it represents deviations from the mean score given a participant’s age, sex and data collector. The GSED was included as a secondary measure of child development as part of global efforts to validate the GSED in different settings. The GSED combines direct observation and caregiver report on developmental milestones (‘combined form’) and is a global tool for measuring children’s developmental outcomes for ages 0–3 years which has been validated in diverse settings^(30,31)^. The GSED is analysed using the GSED Development Score (D-Score) which is converted to the development-for-age z-score (DAZ) for comparison of children’s development across ages^(32)^.

### Infant and young child feeding practices

We measured infant and young child feeding practices using a 24-hour dietary recall^(33)^ and scored the following standard guidelines^(34)^. The minimum acceptable diet was defined as the percentage of children aged 6–23 months who had at least the minimum dietary diversity and minimum meal frequency during the previous day. Minimum dietary diversity was defined as a child aged 6–23 months who consumed at least five out of eight food groups during the previous day. Minimum meal frequency was defined as the percentage of children aged 6–23 months who received solid, semi-solid and soft foods (plus milk feeds for non-breastfed children) a minimum number of times or more during the previous day as appropriate (e.g. two feedings for breastfed children aged 6–8 months, three for breastfed children aged 9–23 months and four times for non-breastfed children aged 6–23 months).

### Early learning in the home environment

We assessed home environment using the Family Care Indicators as used in the UNICEF Multiple Indicator Cluster Surveys^(35,36)^. Early learning is defined as the percentage of children engaged in four or more activities to support learning in the last 3 days, with any adult household member, father or mother out of a maximum of six potential activities. The availability of children’s books is defined as the percentage of children who have one or more children’s books. The availability of playthings was defined as the percentage of children who played with two or more types of playthings out of three possible categories of playthings (manufactured toys or those purchased from a shop, homemade toys or household objects). Inadequate supervision was defined as the percentage of children left alone or under the supervision of another child younger than 10 years of age for more than 1 hour at least once in the last week. A total sum score for the family care indicators was calculated with the aforementioned items, with a maximum possible score of 30. We measured opportunities for early stimulation using a newly developed tool for measuring early learning for the NC Framework^(37)^ and scored it as a sum score out of a maximum of fourteen opportunities for early stimulation in the prior 24 h.

We also collected information on caregivers’ mental health using the Patient Health Questionnaire^(38)^, a brief valid measure of depressive symptoms that allows screening for moderate and severe depression using predefined cut-off scores. Several studies in the region, including the validation of the tool in Mozambique^(39)^, indicate that cut scores between 8 and 11 can be used, depending on the context and measurement needs. In the current study, we used a cut-off score of 9 as a cut-off for elevated symptoms of depression to have high specificity based on the validation study in Mozambique^(39)^.

### Statistical analysis

We used 95 % CI and *P*-values less than 0·05 to identify statistically significant results, unless otherwise indicated. To account for the dependence among observations within a community, we used cluster-robust standard errors, with the community as the independent unit of randomisation. To address the quasi-experimental study design and potential imbalance between the two intervention groups, we used the inverse probability of the treatment weight/propensity score weighting approach. Propensity scores were estimated using a logit model, in which the dependent variable was an indicator of whether the participant was in the ECD intervention arm or not. We selected baseline variables contributing to propensity score estimation based on their relationship to both intervention status and outcomes of interest and a background literature review and knowledge of study implementation guided us. To assess balance, we tested for variable equality across the intervention arms, as well as equality of the predicted propensity score across intervals of the predicted propensity score distribution. Common support was assessed by using standard common support graphs.

To estimate the intervention effect on continuous outcomes, we used multivariable linear models controlling for baseline outcome measurement and baseline variables potentially associated with the outcome based on theoretical background knowledge. To estimate the intervention effect on dichotomous outcomes, we used a multivariable modified Poisson model controlling for baseline outcome measurement as well as baseline variables potentially associated with the outcome based on theoretical background knowledge.

We checked assumptions for normality and homoscedasticity of residuals using Shapiro–Wilk and Breusch–Pagan tests and inspected and corrected for outliers following standard approaches. Analyses were conducted using the Stata software (version 16).

## Results

Of the 962 children included in the baseline evaluation, 875 (90·95 %) had complete matched information at the endline. Losses to follow-up were similar between the intervention and comparison groups (9·87 % and 8·04 %, respectively). Table 2 presents the characteristics of the children, caregivers and households in the intervention and comparison groups. The baseline indicators reflected the overall vulnerability of the target population, with slightly worse results for participants in the comparison group. A larger proportion of caregivers in the comparison group had no formal education than those in the intervention group (44·1 % *v.* 30·3 %). Children in the comparison group were more likely to be girls (58 % *v.* 48 %), to have participated in early stimulation activities (5·8 % *v.* 2·5 %), to have been left alone or under the care of another child (49·5 % *v.* 41·2 %) and to have higher proportions with minimum acceptable dietary diversity (10·1 % *v.* 5·7 %) but fewer achieving less likely to have minimum acceptable meal frequency (13·2 % *v.* 25·4 %). All other characteristics were generally similar, and propensity score weighting achieved a balance between the two groups (Table 2). We also confirmed balance via an overidentification test for covariate balance, *P* = 0·749 (indicating that the null hypothesis that covariates are balanced was not rejected).

**Table 2.** Baseline characteristics of the intervention and comparison participants as observed and after propensity score weighting

	Comparison (*n* 485)		Intervention (*n* 476)		Standardised mean difference		Variance ratio	
	Mean	sd	Mean	sd	Observed^*^	Weighted^†^	Observed^*^	Weighted^†^
Socio-demographic characteristics								
Age of the child (months)	12·5	6·2	11·4	6·0	−0·23	−0·02	1·02	1·03
Age of the child’s mother (years)	27·9	8·0	26·6	7·4	−0·16	0·01	0·82	0·98
Number of persons living in the household	5·2	1·7	5·4	1·9	0·08	0·01	1·24	1·06
	*n*	%	*n*	%				
Percentage of male children	198	40·8 %	249	52·3 %	0·24	0·00	1·05	1·00
Primary caregivers with no formal education	214	44·1 %	144	30·3 %	−0·40	0·01	0·81	1·00
Primary caregivers with primary education or higher	251	51·8 %	286	60·1 %	0·25	−0·02	0·94	1·00
Primary caregivers who can read/write	90	18·6 %	141	29·6 %	0·37	−0·01	1·61	0·98
Primary caregiver married or living with partner	415	85·6 %	410	86·1 %	−0·04	−0·02	1·08	1·04
Children whose father lives in the home	359	74·0 %	364	76·5 %	0·06	−0·02	0·93	1·02
Improved water source	172	35·5 %	186	39·1 %	0·00	0·03	1·00	1·01
Improved sanitation	33	6·8 %	22	4·6 %	−0·05	0·00	0·82	0·98
Improved floor	19	3·9 %	31	6·5 %	0·13	0·02	1·74	1·07
Improved walls	392	80·8 %	375	78·8 %	−0·07	−0·01	1·10	1·02
	Mean	sd	Mean	sd				
Wealth index	−0·1	0·9	0·1	1·1	0·19	0·01	1·45	0·94
Baseline outcome measures								
Total sum score on all five ASQ-3 domains (maximum of 300)	160·3	63·1	175·9	64·1	0·24	0·02	1·03	1·19
Mean Development-for-Age Z-Score (DAZ) on the GSED combined form	−0·2	1·0	0·0	1·0	0·26	0·01	1·01	1·07
Mean number of activities to support learning with any adult (maximum of 6)	1·5	1·3	1·5	1·0	0·02	0·00	0·68	0·71
Mean number of activities to support learning with mother (maximum of 6)	1·1	1·0	1·2	0·9	0·15	0·01	0·84	0·84
	*n*	%	*n*	%				
Children who have one or more children’s books	23	4·7 %	25	5·3 %	0·03	−0·02	1·12	0·95
Children experiencing inadequate supervision	240	49·5 %	196	41·2 %	−0·25	0·00	1·02	1·00
Children aged 6–23 months with minimum dietary diversity	39	10·1 %	21	5·7 %	−0·16	−0·03	0·61	0·91
Children aged 6–23 months with minimum meal frequency	51	13·2 %	94	25·4 %	0·37	0·00	1·89	0·99
Caregivers with elevated symptoms of depression on the PHQ-9	190	39·2 %	169	35·5 %	−0·12	0·03	0·94	1·01

ASQ, Ages and Stages Questionnaire; GSED, Global Scales for Early Development; PHQ, Patient Health Questionnaire.

* Comparisons as observed in the unweighted data.

† Comparison adjusted by propensity score weights derived from child age and sex, baseline development assessment scores, caregiver education, literacy, household water source, sanitation, building material quality, wealth index and number of people in the household to balance the intervention arms across baseline characteristics.

Table 3 presents the estimated intervention effect results as the endline difference in means between the groups for continuous outcomes or the prevalence ratio for binary outcomes. At the endline, we found a statistically significant mean difference between the intervention and control group total scores on opportunities for early stimulation as measured by the total score for the family care indicator (0·36 points; *P* ≤ 0·001) and similar differences for the total activities to support learning (0·30 activities sd; *P* ≤ 0·004).

**Table 3.** Adjusted differences and propensity-weighted adjusted differences for primary outcomes at endline by group

	Endline values						Adjusted differences^*^			Propensity weighted adjusted differences^†^		
	Comparison			Intervention			Mean difference or prevalence ratio	95 % CI	*P*-value	Mean difference or prevalence ratio	95 % CI	*P*-value
	*n*	Mean	sd	*n*	Mean	sd						
Child development												
ASQ-3 communication Z-score	445	−0·03	0·97	428	0·04	1·03	0·03	–0·12, 0·19	0·675	0·04	–0·11, 0·20	0·596
ASQ-3 gross motor Z-score	445	−0·05	1·00	428	0·05	1·00	0·04	–0·13, 0·22	0·641	0·05	–0·12, 0·22	0·553
ASQ-3 Fine motor Z-score	445	0·01	1·01	428	−0·01	0·99	−0·08	–0·25, 0·08	0·309	−0·08	–0·24, 0·07	0·300
ASQ-3 problem-solving Z-score	445	−0·08	0·98	428	0·08	1·01	0·10	–0·06, 0·26	0·213	0·11	–0·04, 0·26	0·161
ASQ-3 personal-social Z-score	445	−0·09	0·99	428	0·09	1·00	0·11	–0·06, 0·28	0·192	0·13	–0·03, 0·29	0·121
ASQ-3 overall Z-score	445	−0·07	1·01	428	0·07	0·99	0·06	–0·13, 0·24	0·542	0·07	–0·11, 0·25	0·439
GSED combined form development for-age Z-score	442	0·07	0·97	428	0·21	0·95	0·05	–0·14, 0·23	0·622	0·05	–0·13, 0·23	0·604
Early learning in the home environment												
Family care indicator total (sum, maximum score of 30)	446	9·95	1·19	429	10·34	1·49	0·37	0·14, 0·60	0·002	0·36	0·14, 0·59	0·001
Child engaged in four or more learning activities with any adult in the household	446	9·9 %		429	10·7 %		1·10	0·73, 1·67	0·643	1·25	0·81, 1·93	0·316
Total early stimulation activities to support learning (maximum score of 14)	446	1·75	1·35	429	2·13	1·25	0·31	0·09, 0·52	0·005	0·30	0·09, 0·50	0·004
Inadequate supervision	446	69·3 %		429	61·8 %		0·88	0·76, 1·03	0·104	0·88	0·75, 1·02	0·086
Caregiver mental health												
Elevated signs of depression (PHQ > 9)	446	28·9 %		429	31·7 %		1·11	0·85, 1·46	0·440	1·09	0·83, 1·44	0·522
Infant and young child feeding practices												
Minimum meal frequency	192	8·9 %		229	14·4 %		1·54	0·87, 2·71	0·136	1·49	0·81, 2·72	0·200
Minimum dietary diversity	192	8·3 %		226	5·8 %		0·80	0·36, 1·77	0·577	0·84	0·37, 1·90	0·680

ASQ, Ages and Stages Questionnaire; GSED, Global Scales for Early Development; PHQ, Patient Health Questionnaire.

* We adjusted for differences in child age and sex, baseline development assessment scores, caregiver education, literacy, household water source, sanitation, building material quality, wealth index and the number of people in the household.

† We adjusted propensity-weighted adjusted differences for the same set of variables and additionally incorporated propensity score weighting to balance the intervention arms across baseline characteristics.

There were no measurable impacts of the intervention on the primary outcomes of ASQ-3 developmental scores (ASQ-3 or GSED), maternal mental health, infant and child feeding practices, availability of playthings in the household, number of activities carried out by the father or inadequate supervision.

## Discussion

In this study, the integrated NC package building upon an ongoing platform of nutrition, sanitation and hygiene community group sessions with caregivers in Nampula, Mozambique, showed some positive results in improving engagement in certain early learning and stimulation activities in the household. Caregiver and child engagement in activities outside the home increased as a result of the intervention. Furthermore, the intervention did not yield any measurable improvements in ECD outcomes. We found no effect of the intervention on improving or worsening meal frequency and dietary diversity, the two measured outcomes for infant and young child feeding practices. This indicates that the integration of ECD content into the nutrition and SH packages did not negatively affect these nutritional outcomes but did not contribute to their improvement. These results are likely driven by challenges both in the design of the integrated programme (e.g. complexity of family needs in the context and limited duration of the intervention) as well as implementation factors (e.g. gaps in training and workforce capacity).

### Design challenges

Delivering evidence-based ECD interventions at scale requires careful adaptation and consideration of entry points, adequate resources and a phased approach for testing and learning^(9)^. Research studies about integrating additional components of nurturing care into existing programmes at the community level have shown mixed results in terms of improvements in ECD outcomes^(6,40)^. In our intervention, we adapted a set of activities previously tested in the Mozambican context^(24)^ to integrate into an existing community-based platform addressing nutrition, sanitation and hygiene, with the overall objective of promoting caregivers’ knowledge and skills regarding early stimulation and responsive caregiving. However, embedding these activities into an existing intervention with several other priority behaviours to change (e.g. feeding and hygiene) could have lessened the overall effectiveness of the intervention, especially in a population with so many vulnerabilities. In addition, our NC intervention involved multiple components, including training the facilitators, delivery of caregiver group sessions, home visits, mass media and existing nutrition, sanitation and hygiene activities. A possible explanation for the limited effect on caregiving practices and the lack of improvements in ECD outcomes is the complexity of the multi-component behaviour change-focused intervention itself in a context such as Nampula, where many unaddressed needs prevail such as the high rates of maternal depression, poverty and limited caregiver education as observed in our study sample that can affect a caregiver’s ability to provide nurturing care^(15)^. Another bundled nutrition and stimulation intervention demonstrated greater improvements in ECD outcomes with greater father involvement, which could have been strengthened in this design^(41)^. Recent evidence further emphasised the implementation challenges of multi-component interventions often resulting in lower impact compared to single-component interventions^(42)^, necessitating greater attention to implementation processes and quality supports.

The timing, intensity and duration of parenting interventions are known to contribute to the impact of these interventions^(43)^. The relatively short duration of our implementation period (one year) may not have allowed sufficient time to produce measurable changes in ECD outcomes, particularly in a setting like Nampula, where families and children experience many adversities, including poverty, neighbouring conflict and climate emergencies. A similar study in Jordan found no effect of a light-touch remote adaptation of the Reach Up and Learn programme integrated with health and nutrition on children’s development^(44)^ and implementation constraints with the community health workforce in India^(16)^ and Madagascar^(17)^ were attributed to the null results of home-visiting programmes. A home-visiting programme in Rwanda demonstrated improvements in child development among families experiencing adversities using a home-visiting approach with two structured booster visits up to six months following an initial 12-week intervention^(45)^. With only four out of twenty-six group sessions focusing on NC spread across several months and challenges in implementing additional play-based activities that were designed to occur in every session^(46)^, we believe that exposure to ECD-specific content may have been insufficient to bring about the desired behavioural changes. Recent evidence also highlights that programmes that have a very strong emphasis on responsive care are more effective^(14)^, and we explicitly covered this practice in only one session and qualitative findings highlighted how this concept in particular was not well understood by the frontline workers delivering this intervention^(46)^.

### Implementation challenges

The implementation and synchronisation of all components involved in a holistic NC intervention require significant coordination and resources, and challenges in maintaining programme fidelity have been reported, even for standardised interventions^(6)^. Our integrated package was delivered through technical assistance to a local non-governmental organisation. We put mechanisms in place for monitoring programme delivery, fidelity and quality throughout implementation and provided technical assistance through ongoing mentorship and support based on learning. These strategies for programme monitoring showed improvements over time based on observation checklists completed during supervision^(25)^ and informed programmatic adjustments over time, such as expanding the training on ECD to a wider range of actors that provided support to the nutrition group leaders to reinforce this programme content. In our study, we were not able to track the actual uptake of the intervention in terms of caregiver exposure to the group sessions specifically targeting the expanded nurturing care practices. However, we cannot discard quality issues, inconsistencies in the frequency of receiving the intervention and other implementation and contextual challenges. Qualitative research alongside this study, published elsewhere, highlighted some quality issues including a reliance on nutrition group leaders lecturing from the manual rather than conducting the interactive activities as intended, which are important for effective behaviour change^(46)^. While the implementation drew from experiences in the Mozambican context^(24)^, additional time for piloting at a smaller scale and refining the implementation approach could have been beneficial.

Training facilitators across the range of knowledge and skills required to effectively deliver NC group sessions can also be challenging. Aligned with the evidence base, our intention was to demonstrate active group practice and feedback; however, the cascade nature of the training may have made it less effective by the time it reached the level of volunteers as was indicated in the accompanying qualitative research.^(46)^ This learning loss was evidenced by relatively lower changes in pre- and post-test scores at the lower level of the cascades, and only 43 % of trainees demonstrated mastery on pre-/post-tests overall^(25)^. Similar challenges in cascade training models were identified during the scale-up of a nutrition programme in Madagascar^(47)^. Techniques for stronger training at the community level or job aids that simplify the work of volunteers will need to be more carefully considered, along with alternative training models to strengthen this or other similar programmes. For instance, Abimpaye et al. found a facilitator for a group radio programme to be as effective as a more intensive programme requiring more in-person skill-building/supervision^(48)^, Ferla et al. showed that looking at videos as part of capacity strengthening (with supervision and mentorship) improves the skills of community health workers^(49)^, and Bond et al. highlighted how effective and routine training, supervision and job aids can prepare non-specialist community workers to deliver ECD programmes^(50)^.

Another reason that may explain the limited impact of our intervention is the insufficient capacity of the community workers and volunteers who delivered the group sessions and other activities. Facilitators’ education level is known to directly influence the fidelity to the programme content and effectiveness of parenting and ECD interventions by shaping their understanding and ability to adequately deliver the programme’s contents^(51)^. Facilitators with lower education levels may have limited knowledge and may struggle to effectively communicate and implement the components of nurturing care and early stimulation interventions^(52)^, exchange knowledge and skills with caregivers and employ appropriate instructional strategies, such as interactive sessions and hands-on activities, to enhance caregivers’ understanding and application of nurturing care and early stimulation practices^(53)^. Literacy rates in communities where our intervention took place are extremely low, and finding literate volunteers in this context will always be a barrier even when that is the recruitment criteria, which provides an additional reason to invest further in training and job aids that can mitigate the absence of some facilitators’ skills and workforce constraints^(48,49)^. A better assessment of workforce capacity is also needed before the start of the programme, along with the alignment of tools and strategies to existing capacities^(52)^. While we designed the programme with the intention of requiring all volunteers to be literate, this was a challenge in practice. Furthermore, we provided all the written tools in Portuguese and delivered the activities in the local language Makhuwa, which is predominantly used orally. This is important learning not only for our programme but broadly for NC interventions in Mozambique, as the current volunteer model is aligned with the government’s nutrition intervention package (Pacote de Intervenções de Nutrição) and is also primarily restricted to written implementation tools in Portuguese^(54)^. This approach is unlikely to be successful without rethinking how to train and support facilitators through feasible and friendly implementation tools and resources, particularly in fragile settings like Mozambique and elsewhere^(55)^. Ensuring adequate time for understanding workforce constraints and capacity to deliver an integrated package at the design and throughout implementation, with timely data for identifying challenges and employing quality improvement strategies, is essential in any NC programme.

This study had some methodological limitations that should be acknowledged. First, the impact evaluation was not a randomised study; we chose integration intervention districts based on convenience. There were some differences between the comparison districts and the intervention districts where we integrated the nurturing care package. While we attempted to adjust for potential socio-demographic differences in the analysis, we cannot fully eliminate potential bias. In addition, the lack of data on attendance and home visits limits our ability to understand the dose of the programme received. Another limitation of this study is the lack of a validated measure for child development outcomes in Mozambique, which may have resulted in a less sensitive measurement of the study’s main outcome. Likewise, while the Family Care Indicators are a valid measure of the quality of the early learning environment, they were designed in the context of global monitoring surveys^(35,36)^ and may not be sensitive enough to detect certain changes resulting from parenting interventions. Lastly, the research team was not blinded to the group assignments, which could have contributed to some bias in their assessments.

In conclusion, this study adds to the literature on the integration of additional components of nurturing care into existing programmes at the community level and highlights important challenges in achieving effective caregiver behavioural changes that can translate into improved ECD outcomes. Our NC intervention was effective in promoting the engagement of children in certain early stimulation and care practices; however, the overall exposure to early learning and responsive care opportunities remained extremely low in the targeted population. We detected no effects at the level of ECD outcomes, warranting the attention of future interventions, perhaps delivered with greater frequency, for a longer period of implementation, and with more robust mechanisms for quality improvement.

## Supporting information

10.1017/S1368980025100554.sm001 de Castro et al. supplementary material de Castro et al. supplementary material

## Supplementary material

For supplementary material accompanying this paper visit https://doi.org/10.1017/S1368980025100554

## References

[1] Black MM , Walker SP , Fernald LCH et al. (2017) Early childhood development coming of age: science through the life course. Lancet 389, 77–90. 27717614 10.1016/S0140-6736(16)31389-7 PMC5884058

[2] Belsky J , Vandell DL , Burchinal M et al. (2007) Are there long-term effects of early child care? Child Dev 78, 681–701. 17381797 10.1111/j.1467-8624.2007.01021.x

[3] Black MM , Walker SP , Attanasio O et al. (2023) Promoting childhood development globally through caregiving interventions. Pediatrics 151, e2023060221B. 10.1542/peds.2023-060221B PMC12952233 37125880

[4] Britto PR & Ulkuer N (2012) Child development in developing countries: child rights and policy implications. Child Dev 83, 92–103. 22277009 10.1111/j.1467-8624.2011.01672.x

[5] Goldfeld S , Woolcock G , Katz I et al. (2015) Neighbourhood effects influencing early childhood development: conceptual model and trial measurement methodologies from the kids in communities study. Soc Indic Res 120, 197–212.

[6] Jervis P , Coore-Hall J , Pitchik HO et al. (2023) The reach up parenting program, child development, and maternal depression: a meta-analysis. Pediatrics 151, e2023060221D. 10.1542/peds.2023-060221D 37125892

[7] Jeong J , Franchett EE , Ramos de Oliveira CV et al. (2021) Parenting interventions to promote early child development in the first three years of life: a global systematic review and meta-analysis. PLoS Med 18, e1003602. 33970913 10.1371/journal.pmed.1003602 PMC8109838

[8] Dulal S , Prost A , Karki S et al. (2021) Characteristics and effects of integrated nutrition and stimulation interventions to improve the nutritional status and development of children under 5 years of age: a systematic review and meta-analysis. BMJ Glob Health 6, e003872. 10.1136/bmjgh-2020-003872 PMC8319976 34321232

[9] Heidkamp RA , Piwoz E , Gillespie S et al. (2021) Mobilising evidence, data, and resources to achieve global maternal and child undernutrition targets and the Sustainable Development Goals: an agenda for action. Lancet 397, 1400–1418. 33691095 10.1016/S0140-6736(21)00568-7

[10] World Health Organization, UNICEF & World Bank (2018) Nurturing Care for Early Childhood Development a Framework for Helping Children Survive and Thrive to Transform Health and Human Potential. Geneva: World Health Organization.

[11] World Health Organization (2020) Improving Early Childhood Development: WHO Guideline. Geneva: World Health Organization. 32200595

[12] Bliznashka L , McCoy DC , Siyal S et al. (2021) Child diet and mother–child interactions mediate intervention effects on child growth and development. Matern Child Nutr 18, e13308. 34905648 10.1111/mcn.13308 PMC8932723

[13] Brown N , Finch JE , Obradović J et al. (2017) Maternal care mediates the effects of nutrition and responsive stimulation interventions on young children’s growth. Child Care Health Dev 43, 577–587. 28480514 10.1111/cch.12466

[14] Keats EC , Das JK , Salam RA et al. (2021) Effective interventions to address maternal and child malnutrition: an update of the evidence. Lancet Child Adolesc Health 5, 367–384. 33691083 10.1016/S2352-4642(20)30274-1

[15] Martin SL , Zongrone AA , Craig HC et al. (2024) Measuring the intangible resources caregivers need to provide nurturing care during the complementary feeding period: a scoping review in low- and lower-middle-income countries. Public Health Nutr 27, e78. 38223942 10.1017/S1368980024000065 PMC10966882

[16] Kirkwood B , Sikander S , Roy R et al. (2023) Effect of the SPRING home visits intervention on early child development and growth in rural India and Pakistan: parallel cluster randomised controlled trials. Front Nutr 10, 11557763. 10.3389/fnut.2023.1155763 PMC10315474 37404861

[17] Galasso E , Weber AM , Stewart CP et al. (2019) Effects of nutritional supplementation and home visiting on growth and development in young children in Madagascar: a cluster-randomised controlled trial. Lancet Global Health 7, e1257–e1268. 31402006 10.1016/S2214-109X(19)30317-1

[18] Grantham-McGregor SM & Walker SP (2023) Early childhood interventions: issues to consider in getting to scale. Pediatrics 151, e2023060221P. 10.1542/peds.2023-060221P 37125888

[19] Lu C , Black MM & Richter LM (2016) Risk of poor development in young children in low-income and middle-income countries: an estimation and analysis at the global, regional, and country level. Lancet Glob Health 4, e916–e922. 27717632 10.1016/S2214-109X(16)30266-2 PMC5881401

[20] UNICEF (2019) Country Profiles for Early Childhood Development: Mozambique. New York: UNICEF.

[21] Global Nutrition Cluster, Mozambique Nutrition Cluster, UNICEF et al. (2022) A Call to Action: Urgent Scale-Up of Coordinated Nutrition Action Needed in Mozambique. Maputo: UNICEF.

[22] National Institute of Statistics (2023) Projected Population by Area of Residence and Sex According to Age, Nampula, 2021. Maputo: National Institute of Statistics.

[23] USAID Mozambique (2020) Transform Nutrition Fact Sheet. Maputo: United States Agency for International Development (USAID).

[24] Jeong J , Bliznashka L , Ahun MN et al. (2022) A pilot to promote early child development within health systems in Mozambique: a qualitative evaluation. Ann N Y Acad Sci 1509, 161–183. 34859451 10.1111/nyas.14718 PMC8978755

[25] USAID Advancing Nutrition (2023) USAID Advancing Nutrition Mozambique: Final Report Fiscal Years 2019–2023.

[26] Prado EL , Larson LM , Cox K et al. (2019) Do effects of early life interventions on linear growth correspond to effects on neurobehavioural development? A systematic review and meta-analysis. Lancet Glob Health 7, e1398–e1413. 31537370 10.1016/S2214-109X(19)30361-4

[27] USAID Advancing Nutrition (2023) Evaluation of an Integrated Nurturing Care Activity to Improve Early Childhood Outcomes in Mozambique: Evaluation Baseline Report. Arlington, VA: USAID Advancing Nutrition.

[28] Squires J , Bricker DD & Twombly E (2009) Ages and Stages Questionnaires User’s Guide. Baltimore, MD: Brookes Publishing.

[29] Martinez S , Naudeau S & Pereira V (2012) The Promise of Preschool in Africa: A Randomized Impact Evaluation of Early Childhood Development in Rural Mozambique. Washington, DC: World Bank.

[30] Cavallera V , Lancaster G , Gladstone M et al. (2023) Protocol for validation of the Global Scales for Early Development (GSED) for children under 3 years of age in seven countries. BMJ Open 13, e062562. 10.1136/bmjopen-2022-062562 PMC9884878 36693690

[31] McCray G , McCoy D , Kariger P et al. (2023) The creation of the Global Scales for Early Development (GSED) for children aged 0–3 years: combining subject matter expert judgements with big data. BMJ Glob Health 8, e009827. 10.1136/bmjgh-2022-009827 PMC9853147 36650017

[32] Weber AM , Rubio-Codina M , Walker SP et al. (2019) The D-score: a metric for interpreting the early development of infants and toddlers across global settings. BMJ Glob Health 4, e001724. 10.1136/bmjgh-2019-001724 PMC6882553 31803508

[33] UNICEF (2018) Multiple Indicator Cluster Surveys: Questionnaire for Children under Five. New York: UNICEF.

[34] UNICEF & World Health Organization (2021) Indicators for Assessing Infant and Young Child Feeding Practices. Geneva: World Health Organization.

[35] Kariger P , Frongillo EA , Engle P et al. (2012) Indicators of family care for development for use in multicountry surveys. J Health Popul Nutr 30, 472–486. 23304914 10.3329/jhpn.v30i4.13417 PMC3763619

[36] Frongillo EA , Basnet S , Halpin PF et al. (2022) Assessment of positive and stimulating home environments for global monitoring. J Child Fam Stud 31, 473–483.

[37] Hentschel E , Siyal S , Al Sager A et al. (2024) The development and validity of the Early Learning Tool for children 0–3-year-old in rural Pakistan. J Glob Health 14, 04241. 39582246 10.7189/jogh.14.04241 PMC11586645

[38] Kroenke K , Spitzer RL & Williams JB (2001) The PHQ-9: validity of a brief depression severity measure. J Gen Intern Med 16, 606–613. 11556941 10.1046/j.1525-1497.2001.016009606.x PMC1495268

[39] Cumbe VFJ , Muanido A , Manaca MN et al. (2020) Validity and item response theory properties of the Patient Health Questionnaire-9 for primary care depression screening in Mozambique (PHQ-9-MZ). BMC Psychiatry 20, 382. 32698788 10.1186/s12888-020-02772-0 PMC7374823

[40] Ahun MN , Aboud F , Wamboldt C et al. (2023) Implementation of UNICEF and WHO’s care for child development package: lessons from a global review and key informant interviews. Front Public Health 11, 1140843. 36875409 10.3389/fpubh.2023.1140843 PMC9978394

[41] Jeong J , Ahun MN , Gunaratna NS et al. (2024) Effects of engaging fathers and bundling parenting and nutrition interventions on early child development and maternal and paternal parenting in Mara, Tanzania: a factorial cluster-randomized controlled trial. J Child Psychol Psychiatry 65, 694–709. 37800367 10.1111/jcpp.13897

[42] Nores M , Vazquez C , Gustafsson-Wright E et al. (2024) The cost of not investing in the next 1000 days: implications for policy and practice. Lancet 404, 2117–2130. 39571590 10.1016/S0140-6736(24)01390-4

[43] Aboud FE , Yousafzai AK & Nores M (2018) State of the science on implementation research in early child development and future directions. Ann N Y Acad Sci 1419, 264–271. 29791728 10.1111/nyas.13722

[44] Global TIES for Children (2023) Lessons and Impacts of a Phone-Based Parenting Program for Syrian and Jordanian Families with Young Children. New York: New York University.

[45] Jensen SK , Placencio-Castro M , Murray SM et al. (2021) Effect of a home-visiting parenting program to promote early childhood development and prevent violence: a cluster-randomized trial in Rwanda. BMJ Global Health 6, e003508. 10.1136/bmjgh-2020-003508 PMC7849888 33514591

[46] USAID Advancing Nutrition (2023) Integrated Nurturing Care Activity to Improve Early Childhood Outcomes in Mozambique: Qualitative Findings Brief. Arlington, VA: USAID Advancing Nutrition.

[47] Weber AM , Galasso E & Fernald LCH (2019) Perils of scaling up: effects of expanding a nutrition programme in Madagascar. Matern Child Nutr 15, e12715. 30748113 10.1111/mcn.12715 PMC7199089

[48] Abimpaye M , Dusabe C , Nzabonimpa JP et al. (2020) Improving parenting practices and development for young children in Rwanda: results from a randomized control trial. Int J Behav Dev 44, 205–215.

[49] Ferla JP , Gill MM , Komba T et al. (2023) Improvement of community health worker counseling skills through early childhood development (ECD) videos, supervision and mentorship: a mixed methods pre-post evaluation from Tanzania. PLOS Global Public Health 3, e0001152. 37276228 10.1371/journal.pgph.0001152 PMC10241410

[50] Bond L , Cheonga F , Byansi W et al. (2024) Exploring nonspecialist preparedness to deliver an evidence-based, family strengthening intervention in Rwanda: a qualitative study. J Behav Health Serv Res 52, 139–154. 39322918 10.1007/s11414-024-09913-3

[51] Yousafzai AK , Rasheed MA , Daelmans B et al. (2014) Capacity building in the health sector to improve care for child nutrition and development. Ann N Y Acad Sci 1308, 172–182. 24571217 10.1111/nyas.12322

[52] Pearson E , Rao N , Siraj I et al. (2023) Workforce preparation for delivery of nurturing care in low- and middle-income countries: expert consensus on critical multisectoral training needs. Child Care Health Dev 50, e13180. 37807967 10.1111/cch.13180

[53] Britto PR , Lye SJ , Proulx K et al. (2017) Nurturing care: promoting early childhood development. Lancet 389, 91–102. 27717615 10.1016/S0140-6736(16)31390-3

[54] Ministry of Health (2019). Nutrition Intervention Package. Maputo: Republic of Mozambique.

[55] Raven J , Wurie H , Idriss A et al. (2020) How should community health workers in fragile contexts be supported: qualitative evidence from Sierra Leone, Liberia and Democratic Republic of Congo. Hum Resour Health 18, 58. 32770998 10.1186/s12960-020-00494-8 PMC7414260

