# Supplementary material for: The impact of an integrated nurturing care intervention to improve early childhood development outcomes in Nampula Province, Mozambique
Source: Public Health Nutr. 2025 Jun 13;29(1):e93. doi: 10.1017/S1368980025100554 (PMC13398206; doi:10.1017/S1368980025100554)
Supplement: de Castro et al. supplementary material [file S1368980025100554sup001.docx]

**Supplementary Information**

Table S1. Adjusted differences and propensity weighted adjusted differences for additional measures of early learning practices at endline by group

|  | **Endline Values** | | | | | | **Adjusted Differences*** | | **Propensity Weighted Adjusted Differences†** | |
| --- | --- | --- | --- | --- | --- | --- | --- | --- | --- | --- |
|  | Comparison | | | Intervention | | | Mean difference or Prevalence Ratio | | Mean difference or Prevalence Ratio | |
| Maternal—Total activities to support learning | 446 | 1.18 | 1.03 | 429 | 1.53 | 1.10 | 0.28 (0.09, 0.46) | 0.004 | 0.27 (0.09, 0.45) | 0.004 |
| Paternal—Total activities to support learning | 446 | 0.26 | 0.59 | 429 | 0.37 | 0.80 | 0.12 (0.02, 0.21) | 0.079 | 0.11 (0.02, 0.20) | 0.090 |
| Other adults—Total activities to support learning | 446 | 0.77 | 1.12 | 429 | 1.01 | 1.19 | 0.25 (0.04, 0.46) | 0.022 | 0.24 (0.04, 0.44) | 0.019 |
| One book or more | 44 6 | 21.3% |  | 429 | 9.3% |  | 0.44 (0.28, 0.68) | <0.001 | 0.48 (0.30, 0.75) | 0.001 |
| Two or more playthings | 445 | 83.6% |  | 429 | 81.1% |  | 0.95 (0.85, 1.07) | 0.423 | 0.94 (0.84, 1.05) | 0.254 |
| **Early learning in the home environment**  **item level results** |  |  |  |  |  |  |  |  |  |  |
| Read books | 446 | 15.2% |  | 429 | 13.8% |  | 0.82 (0.53, 1.28) | 0.385 | 0.80 (0.50, 1.25) | 0.322 |
| Told stories | 446 | 13.0% |  | 429 | 13.5% |  | 1.08 (0.72, 1.60) | 0.717 | 1.09 (0.73, 1.63) | 0.670 |
| Sang songs | 446 | 35.4% |  | 429 | 48.7% |  | 1.27 (1.02, 1.57) | 0.030 | 1.28 (1.03, 1.59) | 0.025 |
| Took child outside | 446 | 69.1% |  | 429 | 85.3% |  | 1.21 (1.09, 1.34) | <0.001 | 1.19 (1.08, 1.32) | 0.001 |
| Played with the child | 446 | 37.2% |  | 429 | 46.9% |  | 1.22 (0.95, 1.56) | 0.118 | 1.25 (0.98, 1.91) | 0.077 |
| Named or  counted objects | 446 | 5.4% |  | 429 | 4.9% |  | 0.94 (0.49, 1.78) | 0.841 | 1.03 (0.55, 1.91) | 0.932 |

*We adjusted for differences in child age and sex, baseline development assessment scores, caregiver education, literacy, household water source, sanitation, building material quality, wealth index, and the number of people in the household.

†We adjusted propensity-weighted adjusted differences for the same set of variables and additionally incorporated propensity score weighting to balance the intervention arms across baseline characteristics.
